# Microglia: Synaptic modulator in autism spectrum disorder

**DOI:** 10.3389/fpsyt.2022.958661

**Published:** 2022-11-17

**Authors:** Cong Hu, Heli Li, Jinhui Li, Xiaoping Luo, Yan Hao

**Affiliations:** ^1^Division of Child Healthcare, Department of Pediatrics, Tongji Hospital, Tongji Medical College, Huazhong University of Science and Technology, Wuhan, China; ^2^Department of Pediatrics, Tongji Hospital, Tongji Medical College, Huazhong University of Science and Technology, Wuhan, China

**Keywords:** autism (ASD), microglia, neurodevelopmental, synapse, synaptic plasticity

## Abstract

Autism spectrum disorder (ASD) is a neurodevelopmental disorder characterized by variable impairment of social communication and repetitive behaviors, highly restricted interests, and/or sensory behaviors beginning early in life. Many individuals with ASD have dysfunction of microglia, which may be closely related to neuroinflammation, making microglia play an important role in the pathogenesis of ASD. Mounting evidence indicates that microglia, the resident immune cells of the brain, are required for proper brain function, especially in the maintenance of neuronal circuitry and control of behavior. Dysfunction of microglia will ultimately affect the neural function in a variety of ways, including the formation of synapses and alteration of excitatory–inhibitory balance. In this review, we provide an overview of how microglia actively interact with neurons in physiological conditions and modulate the fate and functions of synapses. We put a spotlight on the multi-dimensional neurodevelopmental roles of microglia, especially in the essential influence of synapses, and discuss how microglia are currently thought to influence ASD progression.

## Introduction

Autism spectrum disorder (ASD) is a group of neurodevelopmental disorders characterized by impairments in social communication and restricted or repetitive behaviors or interests (1). The worldwide prevalence of ASD has increased, across all 11 Autism and Developmental Disabilities Monitoring (ADDM) sites in the United States, 1/44 has been estimated to have ASD of 8 years old children reported by the American Centers for Disease Control and Prevention (CDC) in 2021, and incidence and median age varied widely from site to site (2). Accumulating evidence has revealed that both genetic (e.g., *de novo* variants, copy number variations, and large deletions) (3) and environmental factors [e.g., perinatal events (4) and maternal obesity (5)] are potential risk factors for ASD (6). Less is known about the physiological pathology of ASD, but it may involve a number of systemic connections, nerves, biochemistry, cellular, and molecular characteristics (5). It has been recognized that complex interactions and combinations of genetic, environmental factors, and immune dysfunction can play a potential role in its development (7, 8). In particular, previous research has highlighted that ASD is an imbalance of central nervous system homeostasis caused by chronic inflammatory responses, which are often accompanied by the activation of microglia (9, 10).

Neurodevelopmental abnormalities in early life are an essential mechanism of ASD development, in which microglia work as critical regulators. Specifically, neurons have been examined to be over-produced than required for the proper function of the development of the cerebral cortex during fetal neurogenesis (11). Microglia probe their micro-environment and correlate with the activity of individual synapses (12, 13). To maintain environmental stability, when neurogenesis is nearing completion, microglia limit the overproduction of neurons by swallowing precursor neural cells. In addition, they shape the connections between synapses through synaptic pruning during postnatal brain development (11, 14). Once neurodevelopment is completed, microglia act as innate immune cells in the CNS by monitoring the microenvironment and becoming activated when irritated by injury, infection, or disease (15, 16). In the early stages of brain development, chronic neuroinflammation could activate microglia, which could affect the length, orientation, and assignment of dendritic spines on neurons, especially excitatory and inhibitory neuron assignment, inducing the impairment of behavioral and cognitive (17) or social communication impairment in ASD (18, 19). For example, it has been observed in mice that increased synaptic density was caused by inhibited microglial autophagy, ultimately leading to decreased sociability (20).

In summary, recent studies have shown that activation of microglia can influence the structure and function of synapses and the process of neurodevelopment. For instance, synaptic defects disrupt the excitatory and inhibitory balance and synaptic pruning, and the accurate mechanisms in the neurodevelopment of microglia in cellular and molecular ASD remain to be fully elucidated. The main idea of this review is that microglial activation is associated with ASD and may have a significant impact on synaptic function. This study will summarize the factors and pathways that may be involved in microglia and synaptic function, thus providing a new understanding of the role of microglia in ASD.

## Origin and function of microglia

As a vital role in regulating brain development, neuronal networks, and injury repair, microglia are the primary cells that maintain defense stability throughout the brain parenchyma (21). Recent studies highlight that microglia are a type of phagocytic cell in the brain that can eliminate entire cells or substructures of cellular, especially synapses both in humans and mice (16, 22).

The origin of microglia is shown concretely in mouse studies. During the first trimester after pregnancy in murine, the fate-mapping analysis revealed that adult microglia derive from C-KIT^+^/CD41^+^ erythromyeloid myeloid progenitors that arise before embryonic day 8 (E8.0) in the developing yolk sac (23). During fetal development, microglia progenitor cells migrate and colonize in the brain before cerebral vascular branching is completed and the blood–brain barrier (BBB) is fully shaped (24, 25). In mice, these precursors migrate into the embryonic brain around E9.5 and are restricted by the fully completed BBB as an autonomous, long-lived cell population that maintains the ability to divide and self-renew throughout the lifespan (26–28).

During neurogenesis of early brain development, excess synaptic connections will be removed to maintain proper connections by synaptic sculpting, which is important for maintaining normal neural function (29). Microglia exert a vital role in regulating immature synapses during development *via*engulfing synaptic structures and synaptic pruning (30). For example, in mice with the defective function of microglia, the spine density and the frequency of miniature excitatory postsynaptic currents were increased (20, 31). Therefore, it could be speculated that the aberrant function of microglia influenced by risk factors of genetics and environment, could lead to neurodevelopmental disorder in ASD.

## The regulation of microglia on inflammation

Nowadays, neuroinflammation has increasingly gained interest as a target to explore the mechanism of neurodevelopment (32). The occurrence and spread of neuroinflammation are closely related to the interaction between microglia and neurons (33). While certain features of closely regulated proinflammatory activity are necessary for healthy neural development, uncontrolled early inflammation may alter the programming of the microglial population itself, thereby perpetuating neuroinflammatory damage produced early in life (34).

Microglia are thought to be “quiescent” but recent evidence suggests that they continuously scan the brain environment and make contact with synapses in the normal brain (29, 35). Microglia are easily affected by environmental high-risk factors, especially in pregnancy and prenatal development. Interestingly, in the early pathological process of many diseases, microglia are rapidly activated, which is characterized by increased somatic cell size and pro-inflammatory cytokines. Reactive microglia could have an important impact on different processes of neuroinflammation. They can not only act directly *via* the abundantly expressed or release the molecules or mediators, such as interleukin-6 (IL-6), interleukin-1β (IL-1β), tumor necrosis factor -α (TNF-α), NO, C-X-C chemokine receptor type 4 (CXCL-4), and toll-like receptors (TLRs) (36), but also indirectly act as catalysts and amplify cellular and molecular responses, influencing neurogenesis, and BBB permeability (37, 38). Microglia activation would lead to abnormal neurogenesis and changes in synapse pruning, then resulting in structural dysfunction of neurons in adults and neuronal–microglia interaction disorders in the subsequent process (29, 39, 40).

Additionally, specific histone marks deposited in genomic regions associated with inflammatory pathways maintain microglia priming and long-lasting memory following initial exposure to inflammatory stimuli in animal studies. In subsequent immune stimulation, aberrant activation of inflammatory pathways in microglia leads to a loss of immune homeostasis (41). Therefore, if there is an immune challenge early in life, microglia with enhanced activation may be left in the brains of offspring (42, 43). This may be an important mechanism by which children exposed to environmental risk factors early in life are prone to neuroinflammation and abnormal synapse formation.

Recent evidence also indicates that the ongoing process of neuroinflammation suffered by children with ASD may come from intestinal microbiota dysfunction, resulting in microglial activation in different brain areas (44). When microglia are activated continuously for a period, mediators will be constantly produced and then lead to the diminution of synaptic connections and neuronal cell death (45).

### Microglia in ASD

Several studies have shown that there are obvious abnormalities of microglia both in the morphological characteristics and the functions in the brains of patients with ASD, which would cause defects in social interaction and communication (46, 47). Brain tissues obtained from 11 patients with ASD have demonstrated microglia were consistently activated in all brain regions, especially in the cerebellum (40). Cerebral cortex of patients with ASD exhibited altered microglial activity as evidenced by morphological changes, including the increased microglial soma size and extension of filopodia (46). In addition, the 18 kDa translocator protein (TSPO) is highly responsive to inflammatory stimulation, which seemed as an *in vivo* marker of microglia activation (48, 49). Recent studies have sought to detect activated microglia in patients using positron emission tomography for the TSPO (48), and shreds of evidence suggested TSPO was broadly increased in different brain regions of ASD, including pre-frontal, temporal, cerebellar, and anterior cingulate cortices (50).

The functional regulation of microglia by many similar genes emphasizes their important roles in ASD. Children with Rett syndrome (RTT) may display many autism-like features, such as impairment of social communication and skills, reduced eye contact as well as restricted interests, and they initially may be diagnosed with autism (51). RTT is attributed to the mutations of the MECP2 gene (52). To better study the pathogenesis of RTT, the MECP2-knockout mice model is often used, which exhibits similar behavioral characteristics to patients with RTT. Previous studies have shown that the autophagy activity of microglia is significantly reduced in MECP2-knockout mice (51). Specific expression of MECP2 in cells of microglia could partially rescue the mouse phenotype in MECP2-knockout mice (29). In addition, disease progression can be prevented by the implantation of bone-derived myeloid cells with microglial phenotype into the brain parenchyma through wild-type bone marrow transplantation into Mecp2-null hosts under irradiation conditioned. However, when cranial irradiation is blocked by a lead shield, and microglial engraftment is prevented, the disease will not be arrested (53). The report confirms the strong neurotoxic activity of glutamate in conditioned culture medium (CM) obtained from Mecp2 deficient microglia, but not astrocytes. Hippocampal neurons treated with CM from Mecp2 deficient microglia showed abnormal development and bead-like dendritic morphology over 24 h, as well as signs of microtubule destruction and damage to postsynaptic glutamate energy components (54). Evidence demonstrates that microglia are involved in pathogenesis with synaptic loss through excessively engulfing, thereby eliminating presynaptic inputs at the end stages of disease (≥P56 Mecp2-null mice) (55). Therefore, the appropriate microglial activity may be critical for the development or maintenance of neuronal circuits.

Some genes have been validated in mouse models and behavioral testing for the possibility of pathogenicity in ASD. For example, evidence shows that exaggerated translation of eIF4E in microglia, but not astrocytes or other neurons, could lead to autism-like behaviors in male mice. Translation of mRNAs requires binding of a translation initiation factor eIF4E with cap (56). Elevating eIF4E translation in males, increases microglial density and size, shifting the function to enhanced phagocytic capacity and altered synapse formation (57). Atg7-deficient microglia resulted in social behavioral defects and repetitive behaviors, characteristic features of ASD. It has shown increasing defects in synaptic refinement, which is significantly correlated with the function of microglia in synaptic pruning (20).

There are several environmental factors that contribute to placental inflammatory histological changes and the production of pro-inflammatory cytokines, including maternal obesity, depression, and smoking (58, 59). For example, the expression of TLR4 mRNA in placental immune and non-immune cells increased 3–9-fold in obese mothers, which correlated with IL-6 expression in placental, leading to microglial activation in offspring (60, 61).

On the one hand, we can find that a variety of ASD-related genes could cause changes in microglia morphology or function, leading to the onset and development of the disease. On the other hand, heterogeneous environmental states, including maternal asthma, gestational diabetes, pre-eclampsia, and air pollutants exposure, are inducing microglial activation and increasing the risk of ASD (62–64). Each proinflammatory state may have multiple mechanisms of action, and one of the important mechanisms is that the activation microglia regulate metabolic stress, oxidative stress, and neuroendocrine mechanisms to affect neural development (65, 66). The assumption is that microglia are particularly important in the etiopathogenesis of genetic and environmental factors in ASD. Therefore, it is essential to improve our comprehension of how microglia affect neural development.

### MIA and microglia activation

Several environmental risk factors increase the risk of ASD, such as lifestyle, prenatal or maternal exposure, including maternal smoking, toxins, gestational diabetes mellitus (GDM), thyroidism alteration, and infections (viral or bacterial) (4, 67). For instance, exposure to maternal GDM diagnosed by 26 weeks of gestation was linked to an increased risk of ASD in offspring in a large, multiethnic clinical cohort of singletons (68). Epidemiological studies reported a significant association between allergic diseases and ASD risk both in maternal and infants, including asthma, eczema, atopic dermatitis, allergic rhinitis, and food allergies (69–71). One study has demonstrated the activation of spinal microglia in adult asthma and atopic dermatitis models (72), whereas another study shows that allergic immune activation in prenatal maternal attenuated microglial activation in rats (73). A meta-analysis by Jiang et al. shows that maternal infections during the first or second trimester of pregnancy, whether bacterial, viral, or otherwise, were associated with a significantly increased risk of ASD in offspring (64). Two meta-analyses conducted by Chen et al. and Wu et al. have found that maternal autoimmune illness is associated with a significant, precise, and consistent increase in the risk of ASD in the offspring (62, 74). Maternal immune dysregulation during gestation is a high-risk factor for autism (75). In cohort studies, infections during pregnancy, such as rubella or influenza viruses, have been shown to have a significant impact on neurodevelopmental processes by causing immune disruptions and cytokine production in the mother (75). Those factors contributing to neurodevelopmental disorders may correlate with altered immune status characterized by microglial activation in various parts of the brain, which is also related to genetic and epigenetic-related effects, neurotransmitter alterations and abnormalities in signaling pathways, and endocrine disruption (4, 20, 76–78).

Maternal immune activation (MIA) models of monkeys and rodents have attracted much attention nowadays. A prenatal polyinosinic polycytidylic acid (poly I:C) model in rhesus monkeys represented increased repetitive behaviors, abnormal communication, and impaired social interactions. First-trimester MIA offspring showed atypical social behavior by inappropriately approaching or remaining in immediate proximity to an unfamiliar animal (79). Extensive work by Dr. Paul Patterson and other highly influential researchers has elucidated part of the mechanisms by which viral infection or viral mimetic MIA in rodents models, which can alter offspring immune function persistently and modify fetal brain development, ultimately lead to the phenotype of autism-like behaviors (42, 80, 81).

Additionally, research in both human and animal models suggested that MIA, during crucial times for neurodevelopment and immune system development, programs the fetal brain and immune system through inflammatory and epigenetic mechanisms (42). MIA models are identified to be related to altered immune status, increased oxidative stress, and an active neuroinflammatory process characterized by microglial activation in various brain regions (82, 83). There are a lot of cytokines that are related to the microglia involved in this progress. MIA could promote the release of pro-inflammatory cytokines, such as IL-6, IL-1β, and TNF-α. In addition, these cytokines may cross the placenta directly into fetal circulation (84) and promote the activation of microglia (85, 86). Then, activated microglia could secrete several cytokines, including IL-6, IL-1β, and TNF-α, to regulate neuronal function and neural plasticity (87).

The vital effect of those cytokines in fetal neurodevelopment of the MIA mice models has been well established (42, 88, 89). IL-6, one of the key factors, was enhanced in the maternal serum, as well as in the placenta and fetal brain in MIA models. Autism-like behavioral changes were not seen in the offspring of IL-6 knockout mice after MIA treatment (90). The combined use of anti-IL-6 antibodies in pregnant mice exposed to poly (I:C) prevented behavioral defects and normalized variations in brain gene expression (90). Wei and his colleagues have confirmed that IL-6 overexpression could impair and facilitate the formation of excitatory synapses between mouse cerebellar (91). IL-1β could inhibit long-term potentiation induction which is involved in reducing synaptic strength (92), as well as modulating memory and learning (93). TNF-α is another immunomodulatory molecule that could produce by glial cells in the CNS. Chronic increase of TNF-α may potentially hinder learning and memory in ASD by scaling up synapses during prolonged activity blockade (94).

Proinflammatory cytokines that cross the disruption BBB (driven by increased proliferation of microglia overexpressing of cyclooxygenase-2 in the fetal brain) may initiate a neuroinflammation cascade, which promotes microglial overactivation and behavioral alterations of ASD life span (95). Immune activation during pregnancy has been considered a potential cause of dysfunctional synaptic pruning and abnormal microglia-mediated neurogenesis (96, 97), which will be discussed soon.

## How microglia act on synapses

As described by Peter in the early 1990s, early brain development was characterized by marked changes in synaptic connections. In the human cortex, synaptic density rises significantly during the first 1–2 years of life, after which competitive and activity-dependent abolition of synapses reduces synaptic connection density by approximately 50% (98). That prolonged pruning process was crucial for the proper development of brain circuits and cognitive functions. In autism, the long-term pruning process was crucial not only in shaping brain circuits by increasing dendritic spine densities but also in the normal development of cognitive function in the process of neural development (99). Besides, To keep a steadily internal environment homeostasis, cells are needed to be equipped with helpers capable of regulating synaptic exfoliation and remodeling, scavenging abnormal proteins, invading pathogens, and damaging tissue fragments (100). Activation of microglia can respond to invading pathogens and local fragments or proteins in a neuroprotective manner (101–103). There are similar pathophysiological characteristics can be identified in the brain between the neurodevelopment diseases and neurodegenerative diseases (104). These actions ensure appropriate signal transduction to regulate clearance processes and transition the response to one of resolution and repair, providing nutritional support for the release of various growth factors (104). Any deficiency in the ability of microglia, either a reduction in cell number or the loss of function, could alter the normal structure and function of brain health (104). Abnormal microglia function plays a constructive role in the early occurrence of those diseases (105, 106). As will be mentioned later, activated microglia cloud induce pathways that impacted synapse function and ultimately neuronal function.

Abnormal cortical circuit development can be found in many neurodevelopmental disorders and neuropsychiatric diseases, which may be a striking feature, including ASD, schizophrenia, and intellectual disability (107, 108). And deregulation of synaptic plasticity in ASD has been approved (109). The function of microglia in neural circuits and synaptic plasticity has received extensive attention (110, 111). In *Tsc2*+*/*− ASD mice and patDp/+ mice that it can be found elevated spined densities in the temporal cortex and cerebellum and defected adolescent pruning (47, 112). In neurons, axon guidance, vesicle release, dendritic spine structure, spine pruning, and synaptic plasticity were closely related to microglia function (113). Microglia dysfunction impaired synaptic pruning and led to deficits in social behavior (20). For example, microglia were found to play an important role in the development of synaptic plasticity. Atg7 is necessary for the formation of autophagic vesicles to transport substances to lysosomes. Atg7-deficient mice showed autism-like behaviors and increasing dendritic spine density. Atg7-deficient microglia co-cultured with neurons showed defective synaptosome breakdown, confirming that microglia malfunction causes aberrant synaptic pruning (20). The spine numbers were also increased in mice deficient in microglial Atg7 (114, 115). Pten^m3m4/m3m4^ mice without nuclear PTEN have shown autism-like behaviors and active microglia with increased Iba1 and C1q expression, which identifies microglial activation has an etiological role in ASD *via* regulating synaptic pruning (116).

In the mammalian cerebral cortex, there is a dynamic process of simultaneous formation and elimination/pruning in postnatal synaptic development (117). There are excessive synapses produced in early life which is critical for the remodeling of neural circuits. Synaptic pruning is continuous from childhood to adolescence, and alters the density of dendritic spines peaks in early childhood, followed by a sharp decline to adult levels in later life, culminating in the formation of normal neural network relationships (118). Therefore, we list a number of elements and mechanisms that demonstrate the significance of microglia in synaptic function (Table 1).

**Table 1:** Related mechanisms in microglia acting on synapses.

	**Normal function**	**Dysfunction in microglia**	**Reference**
GABA	Promoting neural stem cell proliferation, neuron migration, axon growth, synapse formation, and circuit perfection	1. Microglia depletion or GABA receptors ablation may lead to superabundant synapses 2. Blocking GABA transport abolished stimulation-induced microglial responses 3. Activated microglia impaired synapse formation and synaptic GABA release	(119–123)
Glutamate	Glutamate could be divided into three types: NMDAR, AMPARs, mGluRs, co-regulating E/I balance with GABA, and altering synaptic plasticity	1. AMPARs and NMDARs function damage could be found in the depletion of microglia 2. Expression of mGlu1 receptors is increased in the ASD models 3. Microglia expression neuroligins alter glutamate receptor function leading to the density of excitatory synapses increased, which would lead to E/I imbalance	(57, 124–132)
BDNF	Promoting neuronal survival, growth, and differentiation, promoting neurotransmitter releasing, impacting synaptic and structural plasticity	1. BDNF acting on microglia could increase phosphorylation of neuronal tropomyosin-related kinase receptor B, mediating synaptic plasticity 2. BDNF level could be altered by the pro-inflammatory cytokine expressed by activated microglia	(133–141)
TREM2-DAP12	Modulating microglia phagocytosis and the overall fitness of microglia	• Expression of TREM2 in microglia decreased the ability of phagocytic membrane fragments and increased proinflammatory cytokines	(107, 108, 135, 142–144)
CX3CL1–CX3CR1	Regulating neurons maturation, promoting microglia migration and proliferation, recognition of synapses engulfment, shaping synaptic plasticity	1. In Cx3cr1-deficient mice, decreased LTP could correlate with synaptic function. 2. In knockout of the Cx3cr1 gene of microglia, it will cause microglia transient decrease, leading to synaptic pruning defects	(11, 108, 145–149)
C3 and C4	Involving in synaptic pruning	• Inhibition of C3 or C4 reduces the number of phagocyte microglia and the degree of early synaptic loss	(150–156)

### Related mechanisms of microglia in ASD

#### GABA

Gamma-aminobutyric acid (GABA), an essential neurotransmitter, could promote neural stem cell proliferation, neuron migration, axon growth, synapse formation, and circuit perfection. In addition, as the major inhibitory neurotransmitter in the mature brain, it also could mediate the transmission of nerve signals (119). Emerging evidence suggested that it is a common pathophysiological phenomenon that imbalance between inhibitory and excitatory transmission of neurons in children with ASD (157). An imbalance of E/I in neural circuits has been postulated as a key neurobiological characteristic of ASD (158). Notably, evidence has shown abnormal expression of GABA receptors in the postmortem brain of patients with ASD (159).

Microglia express receptors for sensing synaptic activity, such as GABA and glutamate (120). GABA can initiate a transcriptional synaptic remodeling program within microglia to shape inhibitory connections without affecting excitatory synapses. Loss of function of GABA receptors in microglia disrupts this process and leads to abnormal behavior (121). Microglia depletion or GABA receptors ablation may lead to superabundant synapses (121). Co-culture with activated microglia impaired synapse formation and synaptic GABA release of induced pluripotent stem cells (iPSCs) (122). In mice, blocking GABA transport abolished stimulation-induced microglial responses (120). Furthermore, activation of the toll-like receptors 4 (TLR4) signaling pathway following maternal LPS exposure induced the abnormal activation of microglia, leading to lower social and exploration behavior, and more repetitive behaviors in offspring (160, 161). LPS can activate microglia through TLR4 and release the proinflammatory cytokines, IL-1β, which subsequently inhibits GABA receptor activity at postsynaptic sites and reduces GABA synthesis at presynaptic sites (123, 162).

#### Glutamate

The underlying process affecting aberrant synaptic plasticity is that E/I imbalance can be mediated not only by GABA but also by Glutamate (Glu) (124). Dysregulation of glutamatergic and dysconnectivity of functional in ASD are arising from the alterations of glutamatergic and GABAergic which changes brain functional connectivity and ultimately contribute to behavioral disabilities (163–165).

There are three main types of glutamate receptors called n-methyl-d-aspartate receptors (NMDARs), α-amino-3-hydroxy-5-methyl-4-isoxazole propionic acid receptors (AMPARs), and metabotropic glutamate receptors (mGluRs) (125). NMDARs mediated synaptic overexpression led to the amplification of postsynaptic plasticity of neurons (126). Established evidence suggested that activity-dependent AMPARs insertion and removal from the postsynaptic membrane played an important role in the long-term plasticity of excitatory synaptic transmission (127). Much evidence supported that both NMDARs and AMPARs have been closely associated with ASD children (128). Moreover, the depletion of microglia could damage the function activity both in AMPARs and NMDARs (129).

Decreased glutamate concentrations in the cortical were linked with the severity of social impairments (125). Nlgn3KO mice, exhibiting observably autism-like behaviors, have markedly increased mGlu1 receptor expression in the brain, which is resulted from mGlu1 receptor blockade (130–132). RNA-Seq data were indicating that microglia from male MG^4E^ mice (expressed highly eIF4E) upregulated the expression of several cytokines (C-X-C motif chemokine 10 (CXCL10), CXCL16, IL-1β). These cytokines might upregulate microglia expression neuroligins through post-translational modification, alter glutamate receptor function, and lead to mEPSCs amplitude increasing (57). Finally, E/I imbalance was often affected by an increase in excitatory synaptic function, as well as an increase in excitatory synapses density (166), which resulted in autism-like symptoms (57).

#### BDNF

Brain-derived neurotrophic factor (BDNF) is the major principal neurotrophic cytokine in the CNS. It contributes to the development of the prenatal and postnatal brain, which can not only promote neuronal survival, growth, and differentiation (167) but also profound impact on synaptic and structural plasticity (133). The near meta-analysis showed that higher peripheral BDNF was consistent with several neurological and psychological theories on the etiology and core manifestations of ASD (167).

BDNF acting on microglia could increase the phosphorylation of neuronal tropomyosin-related kinase receptor B, an important mediator of synaptic plasticity (134). BDNF is occurred both at presynaptic and postsynaptic sites, promoting neurotransmitter releasing, promoting the function of ion-transmitters and NMDARs, and accelerating the potency NMDARs and ion-transmitters (135). Overall, BDNF appeared to enhance excitatory synapses and weakened inhibitory synapses, leading to an imbalance of E/I (45). Similar conclusions were reached in gene-depleted microglia BDNF (134).

BDNF was found to be highly expressed in children with ASD (136), BDNF may lead to increased synthesis of synaptic proteins associated with autism and participate in the development of autism (137), which may enhance synaptic plasticity (138)or increase the density of dendritic spine (139). Besides, BDNF mRNA levels can be decreased by increased levels of the pro-inflammatory cytokine IL-1β, which is mainly expressed by activated microglia (140).

Together, these findings showed a crucial link between higher BDNF levels in the neurons of people with ASD (133), inducing synapse formation, altering functional connectivity and myelination, and modulating behavioral performance (141).

#### TREM2–DAP12

DNAX-activating protein of 12 kDa (DAP12) is a signaling protein expressed by a variety of cells for general function, which act in immune responses (108). Triggering receptors expressed on myeloid cells (TREMs) are a family of cell surface receptors expressed broadly on myeloid cells, TREM2 stimulates the protein tyrosine kinase ERK *via* DAP12, and TREM2-DAP2 is a complex expressed exclusively in microglia in the CNS (142), sustaining many transcriptional programs (108, 143). TREM2 is anchored on the surface of microglia and interacts with DAP12 to initiate signal transduction pathways that promote microglia activation, apoptosis, phagocytosis, and survival. The defective TREM2-DAP12 function is identified to be involved in the pathogenesis of ASD (144).

Interestingly, TREM2 is highly expressed in resting (unstimulated) microglia and downregulated after LPS or IFN-γ inflammatory stimulation (135). Among the many complex processes that happened during brain development, the TREM2-DAP12 axis is critical for maintaining central nervous system tissue homeostasis by modulating microglia phagocytosis and the overall fitness of microglia throughout their life cycle (107). The expression of TREM2 in microglia decreased the ability of phagocytic membrane fragments and increased the gene transcription of proinflammatory cytokines (107). Notably, long-term circuit hyperexcitability and decreased functional connectivity were observed in Trem2 (KO) mice, which displayed modifications in social behavior and repetitive behaviors resembling the phenotype of autism in humans (144).

#### CX3CL1–CX3CR1

The CX3CL1 (fractalkine)-CX3CR1 signaling pathway is the most important communication channel between neurons and microglia. Neurons' expression of the CX3CL1 and connection with CX3CR1 expression on the surface of microglia, their combined interaction plays an important role in regulating neurons maturation and function (108).

CX3CR1, encoded by the Cx3cr1 gene, is a Gi-protein coupled receptor that is mainly expressed by microglia in the CNS (168). CX3CL1 is mainly expressed in neurons, combined with CX3CR1 (169), and is thought to be a shutdown signal, maintaining microglia in a resting situation (168, 170). On the one hand, soluble fractalkine might act to promote microglia migration into the brain or proliferation during development; on the other hand, tethered or locally released fractalkine might be critical for microglia recognition of synapses before or during engulfment, in which case the density of microglia might be normal but the efficiency of engulfment might be reduced (11).

In the Cx3cr1-deficient mice, a transient decrease in microglia density was founded at different stages of development (Postnatal days 8, 15, 28), which impaired its ability to phagocytose synaptic material (11). In the mice hippocampus, altered phagocytosis causes a transient increase in dendritic spine density and a raising in Psd95 protein detected in microglial activation (11). Immature connections enhanced long-term depression (LTD), and impaired function of excitatory synaptic networks during the development of the hippocampal, especially the postnatal area, leads to altered neural function (145). Both young and adult mice exhibited deficits in social communication and repetitive behaviors increasing, which were closely linked to decreased functional connectivity and reduced synaptic transmission in the CNS (11, 146). CX3CL1 can also affect synaptic plasticity. For example, upregulation of CX3CL1 expression in the hippocampus is associated with processes that shape memory-related synaptic plasticity (147). Yet, a detectable reduction in long-term potentiation (LTP) correlates with synaptic function in Cx3cr1-deficient mice (148). In conclusion, once the microglia-specific Cx3cr1 gene was knocked out, it resulted in a transient decrease of microglia, defects in synaptic pruning, and autism-like behavior (146).

The synapses morphogenesis was relying on the signal of CX3CL1-CX3CR1 induced by microglia (11), and this signaling is also a key pathway for neuron-microglia interactions (149).

#### C3 and C4

Microglia play vital roles in eliminating excess synapses through the process of synapse pruning (150). This process requires involving of complement, for instance, labeling of unnecessary synapses with complement 3 (C3), and subsequent recognition of C3 by microglia C3 receptors (151).

Complement 4 (C4) promotes the activation of C3, making C3 firmly attached to its target, mediating the phagocytosis of the labeled target by microglia (152). Therefore, C4 deficiency led to synaptic elimination aberrant (153). In a complement-focused study, patients with ASD have significantly higher degrees of C4B variant deficiency, compared with controls (154), it could also find that C1q, C3, and C4 mRNA levels were visibly reduced in the prefrontal cortex (155). Inhibition of C1q, C3, or CR3 reduces the number of phagocyte microglia and the degree of early synaptic loss (156).

Collectively, interactions between complements may affect synaptic recognition and phagocytosis by microglia. Future research on C4 risk variants and other complementary systems is needed to uncover the possible mechanisms for psychiatric disorders and may provide new ideas for treatment (22).

## Conclusion

**Figure 1:**
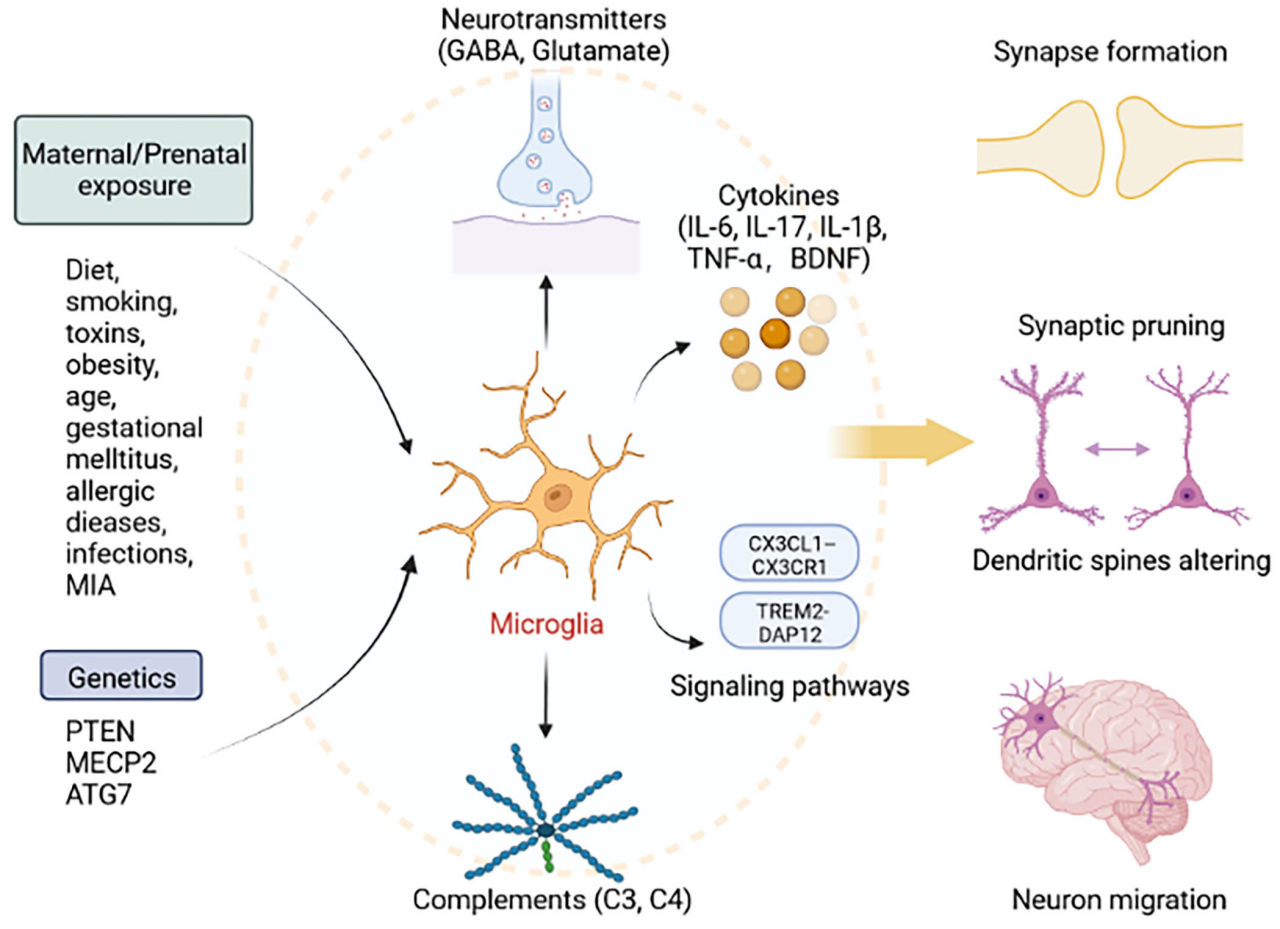
Roles of microglia in ASD. Exposure to factors during pregnancy, including diet, smoking, toxins, obesity, age, gestational mellitus, allergic diseases, infections, MIA, and genetic factors, including mutations in PTEN, MECP2, and ATG7, can lead to abnormal microglial function. Microglia can secrete cytokines (IL-6, IL-17, IL-1β, TNF-α, and BDNF), neurotransmitters (GABA, Glutamate), and complements (C3 and C4), and activate signaling pathway (TREM2-DAP12 and CX3CL1–CX3CR1). Thus, activated microglia lead to the abnormal development of synapse formation, synaptic pruning, dendritic spines altering, and neuron migration. This figure was created with BioRender.com.

Taken together, functional mechanisms of microglia affecting ASD appear to be increasing. Evidence supports a role for microglia influenced by genetics and environment is crucial in ASD pathogenesis. According to the similar pathophysiological characteristics which have been identified in both neurodegenerative diseases and ASD, we assume that microglia have irreplaceable roles in shaping neuronal connectivity by regulating synaptic plasticity, and E/I balance. We summarize the essential cytokines and pathways related to microglia dysfunction which leads to impairments of behaviors. Suggested microglia are essential for neurodevelopment in ASD, far beyond current research understanding (Figure 1). We believe that exploring the mechanisms of action of microglia in ASD is critical for future prevention and treatment.

## Author contributions

CH and JL made manuscript planning and structuring. HL and CH did literature searching and drafted the manuscript. YH and XL revised the manuscript and edited the final manuscript. All authors have read and approved the final version of the manuscript.

## Funding

This work was supported by Huazhong University of Science and Technology Emergency Technology Research Project Response to COVID-19 (Grant Number 2020kfyXGYJ020) and Key Project of Independent Innovation Research Fund of Huazhong University of Science and Technology (Grant Number 2017KFYXJJ100).

## Conflict of interest

The authors declare that the research was conducted in the absence of any commercial or financial relationships that could be construed as a potential conflict of interest.

## Publisher's note

All claims expressed in this article are solely those of the authors and do not necessarily represent those of their affiliated organizations, or those of the publisher, the editors and the reviewers. Any product that may be evaluated in this article, or claim that may be made by its manufacturer, is not guaranteed or endorsed by the publisher.
